# Supplementary material for: Towards building multilingual language model for medicine
Source: Nat Commun. 2024 Sep 27;15:8384. doi: 10.1038/s41467-024-52417-z (PMC11436924; doi:10.1038/s41467-024-52417-z)
Supplement: Supplementary file 1 — Supplementary Information [file 41467_2024_52417_MOESM1_ESM.pdf]

Towards Building Multilingual Language Model for Medicine (Supplementary Material)

Pengcheng Qiu[*,1,2], Chaoyi Wu[*,1,2], Xiaoman Zhang[1,2], Weixiong Lin[1,2], Haicheng Wang[1], Ya Zhang[1,2], Yanfeng Wang[1,2,†] and Weidi Xie[1,2,†]

[1]Shanghai Jiao Tong University [2]Shanghai AI Laboratory

https://github.com/MAGIC-AI4Med/MMedLM

A Used Prompts

In this section, we introduce the prompts used in our methodology and experiments.

A.0.1 Zero-shot prompt

For models such as GPT-3.5 and GPT-4, we employ specific instructions to steer the model's response to questions. To prompt the model for a straightforward correct answer, we use the following directive:

> You're a {language} doctor, make a choice based on the question and options. You need to answer the letter of the option instead of answering the entire option or anything else. Options may not be unique.

When we aim for the model to provide not just the correct answer but also its corresponding rationale, we utilize a different instruction:

> You're a {language} doctor, make a choice based on the question and options in {language}. You should solve this step-by-step. You must first give the reason in {language} for your choice ends with '[End]'. Then you must give the answer's letter directly again. The template is like 'Reason:... [End] Answer: A, B'

A.0.2 Fine-tuning Prompts

Similar to the prompting technique employed in GPT models, we utilize two distinct types of prompt instructions in our fine-tuning setting so that models can understand whether to output long rationale sentences or not, avoiding confusion when training on the mixture of rationale and multiple-choice data. In detail, for multiple-choice answering, we apply the following prompt:

> You're a {language} doctor, kindly address the medical queries according to the patient's account. Answer with the best option directly.

In contrast, to obtain an answer accompanied by its corresponding rationale, we use a more detailed prompt:

> You're a {language} doctor, kindly address the medical queries according to the patient's account in {language}. Let's solve this step-by-step. You should first give the reason in {language} for your choice. Then you should give the right answer index of the question.

It is important to note that during fine-tuning, we employed both of the aforementioned instructions. This implies that for each sample, the model underwent optimization twice in each epoch, with different instructions and corresponding outputs. During evaluation, the first instruction was used to determine the model's accuracy, owing to its significant convenience in metric calculation and the second instruction is utilized to generate rationales and to assess their similarity with the reference material.

A.0.3 Ranking Prompt

We used the following instructions for rating the rationales with GPT4.

> Please act as an impartial judge and evaluate the quality of the responses provided by six AI assistants to the user question displayed below. You should choose the assistant that follows the user's instructions and answers the user's questions better. Your evaluation should consider factors such as the helpfulness, relevance, accuracy, depth, creativity, and level of detail of their responses. Begin your evaluation by comparing the six responses. Avoid any position biases and ensure that the order in which the responses were presented does not influence your decision. Do not allow the length of the responses to influence your evaluation. Do not favor certain names of the assistants. Be as objective as possible. Your output is the ordering of these six models from high to low. Output your final verdict from high to low by strictly following this format: Model A, Model B, Model C, Model D, Model E, and Model F.

A.1 Rationale Generation Prompt

We used the following instructions for generating rationales for each sample with GPT4.

> You're a {language} doctor. Analyze the reasons behind choosing this particular option in 100 words for the following question in {language}.
> ###Question: {question} ###Options: {options} ###Answer: {answer_id}
> ###Rationale:

, where '{language}' stands for the language of the question, '{question}' stands for the question stem, '{options}' stands for the question options and '{answer}' stands for the ground-truth answer.

B Baseline Summarizing

In this section, we conclude all the compared models in Supplementary Table 1. We list out the model size, targeting languages and used training data. For data, we further distinguish them as pre-training data or instruction-tuning data. We use "{model}+{data}" format to denote what base model it used and what data they extra used.

C MMedC Details

C.1 Settings for Filtering Content

In this section, we present the parameters employed for data filtering in Supplementary Table 2, which are meticulously configured based on the CulturaX dataset. Our parameterization adopts a relatively stringent criterion aimed at procuring high-quality data.

C.2 Data Statistics

We give the details about the composition of MMedC in Supplementary Table 3, including the amount of data coming from different sources for each language.

D MMedBench Details

We give a detailed statistic of the composition of MMedBench in different languages in Supplementary Table 4.

Supplementary Table 1 | A summary table of all our compared existing LLMs. Generally, we split them into three parts, *i.e.*, Close-source LLMs, General Open-source LLMs and Biomedical Open-source LLMs. We report the release time, model size and training data and targeting language types in the table. For training data, we distinguish out whether they further split the data into pre-training data and instruction tuning data because different models may have carried out both steps or only one of them. For Biomedical LLMs since all models are based on a general model and add extra biomedical data for further training, we use "{model}+{data}" format to denote what base model it used and what data they extra used. In table, we use "Unknown" to denote the step exists but the details are confidential and "-" to denote the step is not involved.

Model	Size	Release time	Language Types	Training Data	
				Pre-training Data	Instruction Tuning Data
**Close-source LLMs**					
GPT-3.5	Unknown	2022.11	Multilingual	Unknown	Unknown
GPT-4	Unknown	2023.3	Multilingual	Unknown	Unknown
Flan-PaLM	540B	2022.12	Multilingual	780B	1836 NLP Tasks
MedPaLM 2	Unknown	2023.5	Multilingual	Unknown	Unknown
Gemini-1.0 pro	Unknown	2024.1	Multilingual	Unknown	Unknown
**General Open-source LLMs**					
BLOOMZ	7B	2023.5	Multilingual	341B	83 Multilingual NLP Tasks
InternLM	7B	2023.7	Multilingual	Unknown	Unknown
Llama 2	7B	2023.7	English	2T	Unknown
Mistral	7B	2023.10	English	Unknown	Unknown
InternLM 2	7B	2024.2	Multilingual	Unknown	Unknown
Llama 3	8B	2024.4	English	15T	Unknown
**Biomedical Open-source LLMs**					
ChatDoctor	7B	2023.3	English	LLaMA	100K Healthcare Conversation Samples
MedAlpaca	7B	2023.4	English	LLaMA	99K Medical QA Samples
PMC-LLaMA	13B	2023.9	English	LLaMA + 4B Medical Book Tokens†	513K Medical QA or Conversation Samples
MEDITRON	7B	2023.11	English	Llama 2 + 46.7B Medical Paper tokens	367K Multiple-Choice QA Samples
BioMistral	7B	2024.2	Multilingual	Mistral + 3B PMC Paper Tokens	367K Multiple-Choice QA Samples
MMedLM (Ours)	7B	-	Multilingual	InternLM + MMedC	-
MMedLM 2 (Ours)	7B	-	Multilingual	InternLM 2 + MMedC	-
MMed-Llama 3 (Ours)	8B	-	Multilingual	Llama 3 + MMedC	-

* Since all the English benchmarks share an official test set, we directly use the scores reported in their original papers for other models. For MedAlpaca, GPT-4, GPT-3.5 and Llama 3, the scores are based on Open Medical-LLM Leaderboard [1]

† In PMC-LLaMA, in addition to book tokens, the authors also sampled a small amount of academical papers as well as the natural corpus to do the mixing for pre-training. For simplicity, in table we only list the mian parts, *i.e.*, 4B book tokens.

Supplementary Table 2 | Parameter Settings for Data Filtering. Given the extensive dataset within CulturaX, the configuration of filtering parameters is designed to prioritize quality over quantity.

Language	Medical Keyword Count	Keyword Density	Remain Text(%)	Tokens
English	5	4%	2.29%	6.56B
Spanish	4	4%	0.92%	3.98B
French	4	4%	0.50%	1.90B
Russian	4	2%	0.48%	1.29B
Chinese	5	5%	1.33%	3.34B
Japanese	5	5%	1.13%	1.93B

Supplementary Table 3 | Corpus statistics. The Multilingual Medical Corpus (MMedC) boasts a substantial size of 105GB, comprising over 25 billion tokens. The dataset is categorized into several types by sources: FData represents filtering-based data, OCRData denotes Optical Character Recognition (OCR)-based data, CData refers to data obtained through web crawling, and OSData signifies data from open-source datasets. Furthermore, we distinguish between HQData (high-quality data) which has undergone human verification, and UQData (unsure-quality data), which has not. The TotAmt (Total Amount) quantifies the total token count for each language, with 'Billion tokens' serving as the unit of measurement.

Language	Family	Filtering Content	Textbooks	Websites	Small-scale Dataset	HQData	UQData	TotAmt
English	Indo-European	6.56	4.00	0.00	0.00	4.00	6.56	10.56
Spanish	Indo-European	3.98	0.31	0.05	0.02	0.37	3.98	4.35
French	Indo-European	1.90	0.02	0.00	0.17	0.20	1.90	2.10
Russian	Indo-European	1.29	0.40	0.00	0.00	0.40	1.29	1.69
Chinese	Sino-Tibetan	3.34	1.21	0.00	0.19	1.40	3.34	4.74
Japaneses	Sino-Tibetan	1.93	0.00	0.10	0.01	0.12	1.93	2.05

Supplementary Table 4 | The detailed statistic of MMedBench for different languages. All the token number is reported as a averaged number on all considered cases. We report the train and test statistic number separated as "train/test" in the table. "Question Type" represents the percentage of multiple choices or single choices among on train r test cases.

Language	Number of Samples	Token Number			Question Type	
		Question	Option	Rationale	Multiple Options	Single Option
English	10178/ 1273	181/ 187	38/ 38	208/ 199	0%/ 0%	100%/ 100%
Chinese	27400/ 3426	41/ 42	36/ 36	214/ 209	0%/ 0%	100%/ 100%
French	2171/ 622	27/ 31	71/ 67	207/ 195	72.59%/ 48.39%	27.41%/ 51.61%
Spanish	2657/ 2743	34/ 37	68/ 58	173/ 167	0%/0%	100%/ 100%
Russian	1052/ 256	20/ 20	6/ 6	177/ 173	0%/0%	100%/ 100%
Japanese	1590/ 199	170/ 169	53/ 51	231/ 224	14.28%/ 19.60%	85.72%/ 80.40%

Supplementary Table 5 | Accuracy across languages on ablation study.

Method	English	Chinese	Japanese	French	Russian	Spanish	Avg.
			MMedLM				
Baseline (InternLM)	42.5	65.56	30.15	24.28	55.08	42.38	43.33
Baseline (InternLM)	44.07	64.62	37.19	24.92	58.20	44.97	45.66
$MMedLM_{en}$	49.02	66.35	39.20	29.58	54.30	45.84	47.38
$MMedLM_{ocr}$	49.73	**70.61**	35.18	31.03	69.53	51.24	51.22
MMedLM	**49.88**	70.49	**46.23**	**36.66**	**72.27**	**54.52**	**55.01**
			MMedLM 2				
Baseline (InternLM 2)	53.02	77.70	46.23	37.78	65.62	56.67	56.17
Baseline (InternLM 2)	57.27	77.55	47.74	41.00	68.36	59.59	58.56
MMedLM 2_{en}	60.80	77.58	45.73	39.55	66.41	59.63	58.28
MMedLM 2_{ocr}	**62.61**	**80.41**	54.27	46.14	77.34	65.83	64.43
MMedLM 2	61.74	80.01	**61.81**	**52.09**	**80.47**	**67.65**	**67.30**
			MMed-Llama 3				
Baseline (Llama 3)	61.51	73.99	43.22	43.73	69.53	60.36	58.72
Baseline (Llama 3)	63.86	78.23	48.24	50.80	71.48	64.15	62.79
MMed-Llama 3_{en}	64.15	77.95	45.73	44.21	71.48	64.82	61.39
MMed-Llama 3_{ocr}	64.94	79.13	48.74	51.93	73.83	67.80	64.40
MMed-Llama 3	**66.06**	**79.25**	**61.81**	**55.63**	**75.39**	**68.38**	**67.75**

E Detailed Experiment Results

In this section, we present detailed results across languages in the ablation study, and rationale evaluation based on different metrics.

E.1 Detailed Accuracy on Ablation Study

Here, we give the detailed results of the ablation study, including both accuracy and rationale evaluation across six languages, as shown in Supplementary Table 5 and 6.

E.2 Different Metrics on Rationale Evaluation

We conducted an in-depth analysis of the scores assigned to the rationales generated by various models, utilizing a range of evaluation metrics. Supplementary Table 7 and Supplementary Table 8 illustrate the performance of each model as measured by the N-gram BLEU metrics under PEFT and full fine-tuning settings respectively. Meanwhile, Supplementary Table 9 and Supplementary Table 10 present the scores achieved by these models under the N-gram ROUGE metrics. Supplementary Table 11 and Supplementary Table 12 further show the BERT score of different models, which is a score calculated by averaging N-gram BERT scores as introduced in the main body.

E.3 Detailed Results on Rating Scores

In Supplementary Table 13 and Supplementary Table 14, we further show the GPT-4 and human rating scores for six languages respectively.

Supplementary Table 6 | Rationale evaluation on MMedB with BLEU-1/ ROUGE-1. The value preceding the symbol '/' represents BLEU-1, while the value following it represents ROUGE-1.

Method	English	Chinese	Japanese	French	Russian	Spanish	Avg.
			MMedLM				
InternLM	46.53/41.86	48.24/48.64	44.89/49.83	41.80/37.95	27.87/21.20	43.42/38.59	42.12/39.68
$\text{MMedLM}_{\text{en}}$	46.96/42.42	48.25/48.92	45.70/50.22	41.61/38.36	26.27/19.88	43.93/39.16	42.12/39.83
$\text{MMedLM}_{\text{ocr}}$	**47.46**/**42.65**	**49.24**/**49.91**	47.61/50.81	44.01/40.42	32.42/24.00	46.00/40.43	44.46/41.37
MMedLM	47.37/41.98	48.68/49.28	**48.95**/**52.34**	**45.39**/**41.41**	**33.24**/**24.67**	**46.68**/**41.35**	**45.05**/**41.84**
			MMedLM 2				
InternLM2	49.48/44.12	51.38/51.58	50.64/53.46	46.73/42.00	32.93/24.05	47.94/41.96	46.52/42.86
MMedLM 2_{en}	50.00/**45.02**	50.81/51.19	50.15/53.11	47.12/43.04	33.06/23.94	47.62/41.64	46.46/42.99
MMedLM 2_{ocr}	49.82/44.75	**51.47**/**52.19**	52.66/55.21	47.84/43.68	36.98/28.17	48.93/43.42	47.95/44.57
MMedLM 2	**50.02**/44.77	51.39/51.78	**54.79**/**57.10**	**49.04**/**45.30**	**37.49**/**28.18**	**50.14**/**44.59**	**48.81**/**45.29**
			MMed-Llama 3				
Llama 3	**48.74**/**43.66**	49.44/49.42	51.96/53.98	47.11/42.49	34.73/25.07	48.59/42.44	46.76/42.84
MMed-Llama 3_{en}	48.68/43.33	49.32/48.95	51.12/53.46	46.24/41.88	35.06/25.61	48.27/42.32	46.45/42.59
MMed-Llama 3_{ocr}	47.49/42.70	49.18/49.45	52.10/54.24	48.34/**43.63**	36.00/26.36	48.48/42.38	46.93/43.13
MMed-Llama 3	47.61/42.47	**49.96**/**49.36**	**52.89**/**55.06**	**47.92**/**42.85**	**36.32**/**26.67**	**48.61**/**43.32**	**47.22**/**43.29**

Supplementary Table 7 | Rationale evaluation of models undergo **PEFT** using n-grams **BLEU metrics**.

Method	Metric	English	Chinese	Japanese	French	Russian	Spanish	Avg.
BLOOMZ	BLEU-1	41.45	43.09	28.79	38.89	22.25	42.36	36.14
InternLM	BLEU-1	41.29	43.47	22.80	30.35	18.24	36.32	32.08
LLaMA 2	BLEU-1	44.72	42.69	45.58	42.93	31.75	44.22	41.98
ChatDoctor	BLEU-1	44.65	40.88	39.54	40.12	30.95	42.88	39.84
MedAlpaca	BLEU-1	43.59	40.71	37.27	39.82	30.11	42.80	39.05
PMC-LLaMA	BLEU-1	44.98	40.09	38.15	38.89	30.08	43.00	39.20
Mistral	BLEU-1	**48.13**	45.61	43.82	**44.73**	33.62	47.37	43.88
Meditron	BLEU-1	44.26	39.26	36.31	38.73	28.34	42.02	38.15
InternLM 2	BLEU-1	46.87	**47.64**	42.22	41.81	26.78	44.51	41.64
BioMistral	BLEU-1	45.85	43.12	38.76	41.82	27.73	45.52	40.46
Llama 3	BLEU-1	46.33	47.09	46.24	43.13	30.89	47.14	43.47
MMedLM (Ours)	BLEU-1	41.63	44.30	38.61	37.54	19.99	40.79	37.14
MMedLM 2 (Ours)	BLEU-1	47.07	47.15	47.90	43.22	27.81	46.17	43.22
MMed-Llama 3 (Ours)	BLEU-1	46.56	47.12	**48.10**	43.62	**33.92**	**47.67**	**44.50**
BLOOMZ	BLEU-2	14.68	15.03	8.46	14.20	5.04	15.92	12.22
InternLM	BLEU-2	14.79	15.28	7.29	9.92	4.59	11.59	10.58
LLaMA 2	BLEU-2	16.29	14.01	18.34	16.57	9.12	16.20	15.09
ChatDoctor	BLEU-2	17.46	13.39	14.91	15.98	9.25	16.45	14.57
MedAlpaca	BLEU-2	16.81	13.35	13.95	15.85	8.81	16.13	14.15
PMC-LLaMA	BLEU-2	17.87	13.27	14.07	14.94	8.71	16.38	14.21
Mistral	BLEU-2	**20.33**	16.26	17.11	**18.56**	10.56	19.32	17.02
Meditron	BLEU-2	17.39	13.08	12.06	14.47	7.95	14.97	13.32
InternLM 2	BLEU-2	18.91	**18.53**	16.29	15.80	7.22	16.53	15.55
BioMistral	BLEU-2	18.74	15.15	14.06	16.03	6.45	17.55	14.66
Llama 3	BLEU-2	19.20	17.52	19.16	17.41	8.57	19.02	16.81
MMedLM (Ours)	BLEU-2	15.33	16.06	14.69	13.76	5.96	15.12	13.49
MMedLM 2 (Ours)	BLEU-2	19.11	18.45	20.09	17.90	8.92	18.98	17.24
MMed-Llama 3 (Ours)	BLEU-2	18.99	17.59	**21.19**	18.37	**10.76**	**20.03**	**17.82**
BLOOMZ	BLEU-3	6.48	6.83	3.50	6.78	1.94	7.50	5.50
InternLM	BLEU-3	6.64	6.97	3.48	4.42	1.80	4.93	4.71
Llama 2	BLEU-3	7.19	5.91	9.09	8.06	3.74	7.32	6.89
ChatDoctor	BLEU-3	8.37	5.82	7.36	7.92	4.00	7.87	6.89
MedAlpaca	BLEU-3	8.04	5.80	6.69	7.76	3.68	7.63	6.60
PMC-LLaMA	BLEU-3	8.75	5.82	6.81	7.08	3.74	7.82	6.67
Mistral	BLEU-3	**10.35**	7.40	8.57	**9.61**	**4.56**	9.68	8.36
Meditron	BLEU-3	8.34	5.76	5.74	7.02	3.20	6.62	6.11
InternLM 2	BLEU-3	9.12	8.98	8.10	7.69	2.84	7.83	7.42
BioMistral	BLEU-3	9.26	6.76	6.58	7.89	2.28	8.38	6.86
Llama 3	BLEU-3	9.45	8.38	9.85	8.81	3.46	9.53	8.25
MMedLM (Ours)	BLEU-3	6.92	7.48	7.59	6.44	2.33	7.11	6.31
MMedLM 2 (Ours)	BLEU-3	9.21	**8.99**	10.59	9.21	3.87	9.75	8.60
MMed-Llama 3 (Ours)	BLEU-3	9.43	8.64	**11.32**	9.60	4.55	**10.18**	**8.95**
BLOOMZ	BLEU-4	3.25	3.38	1.77	3.26	0.87	4.02	2.76
InternLM	BLEU-4	3.53	3.41	1.87	2.14	0.91	2.49	2.39
Llama 2	BLEU-4	3.72	2.76	4.94	4.06	1.77	3.85	3.52
ChatDoctor	BLEU-4	4.52	2.62	3.99	4.10	1.95	4.37	3.59
MedAlpaca	BLEU-4	4.50	2.72	3.60	3.96	1.74	4.18	3.45
PMC-LLaMA	BLEU-4	4.90	2.73	3.71	3.46	1.76	4.30	3.48
Mistral	BLEU-4	**5.92**	3.75	4.72	5.18	2.16	5.63	4.56
Meditron	BLEU-4	4.59	2.65	2.91	3.55	1.46	3.42	3.09
InternLM 2	BLEU-4	5.07	**4.74**	4.41	4.07	1.25	4.21	3.96
BioMistral	BLEU-4	5.24	3.26	3.56	4.04	0.96	4.63	3.62
Llama 3	BLEU-4	5.44	4.20	5.61	4.62	1.56	5.38	4.47
MMedLM (Ours)	BLEU-4	3.70	3.77	4.27	3.16	1.08	3.87	3.31
MMedLM 2 (Ours)	BLEU-4	5.21	4.71	5.96	5.03	1.93	5.61	4.74
MMed-Llama 3 (Ours)	BLEU-4	5.43	4.56	**6.47**	**5.37**	**2.18**	**5.70**	**4.95**
BLOOMZ	BLEU	9.95	10.29	5.61	9.91	3.19	11.32	8.38
InternLM	BLEU	10.34	10.43	5.33	6.76	2.95	7.79	7.27
Llama 2	BLEU	11.14	9.18	13.46	11.69	5.89	11.18	10.42
ChatDoctor	BLEU	12.50	8.84	10.94	11.30	6.14	11.87	10.26
MedAlpaca	BLEU	12.16	8.93	10.15	11.17	5.76	11.56	9.95
PMC-LLaMA	BLEU	13.07	8.91	10.31	10.28	5.80	11.71	10.01
Mistral	BLEU	**15.10**	11.34	12.69	**13.59**	**6.94**	14.27	12.32
Meditron	BLEU	12.47	8.77	8.71	10.25	5.07	10.13	9.23
InternLM 2	BLEU	13.55	**13.27**	12.05	11.43	4.36	11.76	11.07
BioMistral	BLEU	13.62	10.18	10.09	11.48	3.78	12.56	10.29
Llama 3	BLEU	13.97	12.35	14.47	12.53	5.33	14.06	12.12
MMedLM (Ours)	BLEU	10.66	11.21	11.17	9.51	3.58	10.68	9.47
MMedLM 2 (Ours)	BLEU	13.84	13.16	15.21	13.06	5.73	14.21	12.54
MMed-Llama 3 (Ours)	BLEU	13.85	12.74	**16.13**	13.53	6.91	**14.72**	**12.98**

Supplementary Table 8 | Rationale evaluation of models undergo **full fine-tuning** using n-grams **BLEU metrics**.

Method	Metric	English	Chinese	Japanese	French	Russian	Spanish	Avg.
BLOOMZ	BLEU-1	45.94	48.37	44.71	44.47	29.95	45.91	43.22
InternLM	BLEU-1	46.53	48.24	44.89	41.80	27.87	43.42	42.12
Llama 2	BLEU-1	46.87	46.62	48.53	44.43	33.05	45.96	44.24
ChatDoctor	BLEU-1	47.22	44.66	38.87	44.64	32.19	45.68	42.21
MedAlpaca	BLEU-1	47.33	45.72	45.35	43.78	32.80	45.99	43.49
PMC-LLaMA	BLEU-1	47.33	45.87	44.52	43.80	31.14	46.30	43.16
Mistral	BLEU-1	47.16	48.34	48.80	45.83	34.52	47.55	45.37
Meditron	BLEU-1	47.40	47.93	49.13	45.93	33.65	46.42	45.08
InternLM 2	BLEU-1	49.48	51.38	50.64	46.73	32.93	47.94	46.52
BioMistral	BLEU-1	47.96	49.76	49.73	46.34	34.20	47.57	45.93
Llama 3	BLEU-1	48.74	49.44	51.96	47.11	34.73	48.59	46.76
MMedLM (Ours)	BLEU-1	47.37	48.68	48.95	45.39	33.24	46.68	45.05
MMedLM 2 (Ours)	BLEU-1	**50.02**	**51.39**	**54.79**	**49.04**	**37.49**	**50.14**	**48.81**
MMed-Llama 3 (Ours)	BLEU-1	47.61	49.96	52.89	47.92	36.32	48.61	47.22
BLOOMZ	BLEU-2	17.93	18.70	17.89	18.29	8.58	17.69	16.51
InternLM	BLEU-2	18.76	18.79	18.55	15.89	7.44	15.89	15.89
Llama 2	BLEU-2	18.53	16.98	20.42	17.72	9.40	17.31	16.73
ChatDoctor	BLEU-2	19.50	16.29	15.36	18.25	9.76	17.87	16.17
MedAlpaca	BLEU-2	19.30	16.41	18.48	17.82	9.90	17.91	16.64
PMC-LLaMA	BLEU-2	20.05	16.28	17.67	17.99	9.10	17.68	16.46
Mistral	BLEU-2	18.57	17.97	20.47	19.02	10.48	18.33	17.47
Meditron	BLEU-2	19.86	18.29	20.92	19.16	10.04	18.43	17.78
InternLM 2	BLEU-2	21.20	20.76	22.83	20.14	9.72	19.12	18.96
BioMistral	BLEU-2	18.91	19.59	21.73	19.65	10.45	18.54	18.15
Llama 3	BLEU-2	20.80	19.29	23.48	20.50	10.78	20.26	19.19
MMedLM (Ours)	BLEU-2	19.39	18.84	21.63	19.46	10.13	18.99	18.07
MMedLM 2 (Ours)	BLEU-2	**21.52**	**21.46**	**26.75**	**23.18**	**13.42**	**21.54**	**21.31**
MMed-Llama 3 (Ours)	BLEU-2	20.07	19.78	24.73	20.95	11.92	20.31	19.63
BLOOMZ	BLEU-3	8.39	8.97	8.90	9.15	3.66	8.42	7.92
InternLM	BLEU-3	9.20	9.05	9.56	7.56	3.09	7.24	7.62
Llama 2	BLEU-3	8.89	7.64	10.41	8.78	3.63	7.98	7.89
ChatDoctor	BLEU-3	9.62	7.34	7.74	9.17	4.10	8.63	7.77
MedAlpaca	BLEU-3	9.62	7.66	9.39	8.98	4.16	8.65	8.08
PMC-LLaMA	BLEU-3	10.14	7.27	8.91	8.91	3.88	8.46	7.93
Mistral	BLEU-3	8.67	8.42	10.33	9.65	4.41	8.76	8.37
Meditron	BLEU-3	10.02	8.75	10.81	9.79	4.19	8.98	8.76
InternLM 2	BLEU-3	10.71	10.54	12.41	10.55	4.12	9.31	9.61
BioMistral	BLEU-3	8.96	9.73	11.59	10.14	4.76	9.07	9.04
Llama 3	BLEU-3	10.69	9.32	12.65	10.98	4.54	10.56	9.79
MMedLM (Ours)	BLEU-3	9.55	9.20	11.84	10.24	4.20	9.38	9.07
MMedLM 2 (Ours)	BLEU-3	**11.00**	**11.02**	**15.05**	**12.99**	**6.37**	**11.22**	**11.27**
MMed-Llama 3 (Ours)	BLEU-3	10.21	9.90	13.41	11.11	5.37	10.58	10.10
BLOOMZ	BLEU-4	4.55	4.62	4.87	4.73	1.76	4.58	4.18
InternLM	BLEU-4	5.23	4.82	5.51	3.88	1.55	3.90	4.15
Llama 2	BLEU-4	4.85	3.67	5.94	4.66	1.66	4.28	4.17
ChatDoctor	BLEU-4	5.47	3.65	4.42	4.86	1.96	4.71	4.18
MedAlpaca	BLEU-4	5.43	3.88	5.33	4.83	1.95	4.89	4.38
PMC-LLaMA	BLEU-4	5.79	3.58	5.04	4.52	1.93	4.66	4.25
Meditron	BLEU-4	5.77	4.53	6.09	5.44	2.04	4.99	4.81
Mistral	BLEU-4	4.75	4.30	5.74	5.16	2.18	4.88	4.50
BioMistral	BLEU-4	4.96	5.33	6.76	5.57	2.51	5.05	5.03
Llama 3	BLEU-4	6.41	4.80	7.31	6.19	2.17	6.27	5.53
InternLM 2	BLEU-4	6.13	5.84	7.41	5.83	2.03	5.17	5.40
MMedLM (Ours)	BLEU-4	5.42	5.02	7.09	5.71	1.88	5.39	5.08
MMedLM 2 (Ours)	BLEU-4	**6.41**	**6.20**	**9.15**	**7.70**	**3.30**	**6.63**	**6.56**
MMed-Llama 3 (Ours)	BLEU-4	5.92	5.43	7.79	6.30	2.64	6.17	5.71
BLOOMZ	BLEU	12.80	13.14	13.21	13.06	5.70	12.53	11.74
InternLM	BLEU	13.73	13.46	13.95	11.19	4.97	11.17	11.41
Llama 2	BLEU	13.32	11.44	15.28	12.72	5.83	12.10	11.78
ChatDoctor	BLEU	14.10	11.09	11.49	13.09	6.31	12.81	11.48
MedAlpaca	BLEU	14.05	11.49	13.74	12.99	6.36	12.97	11.94
PMC-LLaMA	BLEU	14.74	11.01	13.22	12.76	6.06	12.77	11.76
Meditron	BLEU	14.61	13.02	15.68	14.02	6.57	13.28	12.86
Mistral	BLEU	13.11	12.65	15.16	13.77	6.90	13.25	12.47
BioMistral	BLEU	13.54	14.23	16.55	14.37	7.23	13.52	13.24
Llama 3	BLEU	15.61	13.67	17.87	15.29	6.92	15.27	14.11
InternLM 2	BLEU	15.44	15.28	17.59	14.90	6.48	13.80	13.91
MMedLM (Ours)	BLEU	14.01	13.69	16.72	14.38	6.36	13.80	13.16
MMedLM 2 (Ours)	BLEU	**15.92**	**15.80**	**20.75**	**17.72**	**9.35**	**16.11**	**15.94**
MMed-Llama 3 (Ours)	BLEU	14.87	14.39	18.75	15.60	7.96	15.21	14.46

Supplementary Table 9 | Rationale evaluation of models undergo **PEFT** using n-grams **ROUGE metrics**.

Method	Metric	English	Chinese	Japanese	French	Russian	Spanish	Avg.
BLOOMZ	ROUGE-1	36.81	45.17	38.09	37.49	15.28	39.22	35.34
InternLM	ROUGE-1	38.05	44.71	37.57	32.14	16.79	34.56	33.97
Llama 2	ROUGE-1	39.34	43.71	49.53	39.29	22.66	39.64	39.03
ChatDoctor	ROUGE-1	40.26	42.80	45.00	39.06	22.84	40.23	38.37
MedAlpaca	ROUGE-1	39.52	42.50	44.69	39.57	22.83	39.64	38.12
PMC-LLaMA	ROUGE-1	40.90	42.95	43.67	38.64	22.45	39.80	38.07
Meditron	ROUGE-1	40.42	42.06	43.34	37.88	21.64	39.06	37.40
Mistral	ROUGE-1	42.80	46.31	48.19	41.07	24.75	**42.83**	40.99
BioMistral	ROUGE-1	41.34	44.75	44.46	39.41	18.80	41.07	38.31
InternLM 2	ROUGE-1	41.66	**49.28**	46.91	38.46	21.71	40.13	39.69
Llama 3	ROUGE-1	41.73	47.44	50.43	40.69	22.22	42.70	40.87
MMedLM (Ours)	ROUGE-1	38.83	46.38	46.90	37.78	21.28	38.77	38.32
MMedLM 2 (Ours)	ROUGE-1	41.51	48.36	52.24	**41.36**	**25.70**	42.64	**41.97**
MMed-Llama 3 (Ours)	ROUGE-1	41.57	47.71	**53.18**	40.97	24.87	**43.32**	41.94
BLOOMZ	ROUGE-2	12.73	16.77	13.43	14.01	4.03	15.66	12.77
InternLM	ROUGE-2	13.08	16.71	13.92	10.80	4.43	12.23	11.86
Llama 2	ROUGE-2	13.63	15.18	21.37	15.06	6.41	15.13	14.47
ChatDoctor	ROUGE-2	15.25	15.10	19.18	15.64	6.82	16.13	14.69
MedAlpaca	ROUGE-2	14.77	15.09	18.86	15.68	6.65	15.83	14.48
PMC-LLaMA	ROUGE-2	15.59	15.37	18.11	14.81	6.66	15.90	14.41
Meditron	ROUGE-2	15.12	15.03	16.28	14.15	6.07	14.93	13.60
Mistral	ROUGE-2	**17.28**	17.60	20.49	17.00	7.72	18.10	16.36
BioMistral	ROUGE-2	16.17	16.86	17.96	15.30	4.38	16.57	14.54
InternLM 2	ROUGE-2	15.95	**20.04**	20.26	14.55	6.00	15.62	15.40
Llama 3	ROUGE-2	16.38	18.79	22.16	16.02	6.32	17.99	16.28
MMedLM (Ours)	ROUGE-2	13.78	17.68	19.75	14.44	6.22	15.27	14.52
MMedLM 2 (Ours)	ROUGE-2	16.02	19.94	23.27	17.01	**8.29**	**18.22**	17.12
MMed-Llama 3 (Ours)	ROUGE-2	16.26	18.81	**24.53**	**17.06**	7.87	**18.92**	**17.24**
BLOOMZ	ROUGE-L	33.28	30.71	26.91	34.01	14.56	35.22	29.11
InternLM	ROUGE-L	34.26	31.02	26.31	29.37	15.76	30.99	27.95
Llama 2	ROUGE-L	34.91	28.40	31.82	35.38	21.02	34.99	31.08
ChatDoctor	ROUGE-L	36.57	28.64	30.86	35.63	21.15	35.98	31.47
MedAlpaca	ROUGE-L	35.88	28.87	30.35	36.08	21.47	35.41	31.34
PMC-LLaMA	ROUGE-L	36.84	28.90	29.43	34.96	20.92	35.35	31.07
Meditron	ROUGE-L	36.38	28.58	29.25	34.24	20.19	34.61	30.54
Mistral	ROUGE-L	**38.57**	31.23	31.49	37.23	23.18	37.90	33.27
BioMistral	ROUGE-L	37.13	30.15	29.79	35.58	17.57	36.36	31.10
InternLM 2	ROUGE-L	37.45	**34.57**	31.70	34.95	20.23	35.78	32.45
Llama 3	ROUGE-L	37.91	32.71	33.36	36.55	20.79	37.86	33.19
MMedLM (Ours)	ROUGE-L	35.08	32.18	32.13	34.19	19.70	34.41	31.28
MMedLM 2 (Ours)	ROUGE-L	37.61	33.52	33.92	37.32	**23.95**	**37.95**	34.04
MMed-Llama 3 (Ours)	ROUGE-L	37.26	33.05	**35.02**	**37.57**	23.37	**38.49**	**34.13**

Supplementary Table 10 | Rationale evaluation of models undergo **full fine-tuning** using n-grams **ROUGE metrics**.

Method	Metric	English	Chinese	Japanese	French	Russian	Spanish	Avg.
BLOOMZ	ROUGE-1	40.51	48.26	48.61	41.05	21.50	40.77	40.12
InternLM	ROUGE-1	41.86	48.64	49.83	37.95	21.20	38.59	39.68
Llama 2	ROUGE-1	41.39	46.57	51.21	40.38	23.24	40.37	40.53
ChatDoctor	ROUGE-1	41.97	45.81	47.95	40.25	23.37	40.71	40.01
MedAlpaca	ROUGE-1	42.31	46.49	49.12	40.41	23.15	40.57	40.34
PMC-LLaMA	ROUGE-1	42.87	46.18	48.44	40.23	22.28	40.68	40.12
Meditron	ROUGE-1	42.85	48.61	52.03	41.37	24.10	41.11	41.68
Mistral	ROUGE-1	41.82	47.91	50.60	40.88	24.68	41.41	41.22
BioMistral	ROUGE-1	42.16	49.33	52.12	41.64	24.27	41.11	41.77
InternLM 2	ROUGE-1	44.12	51.58	53.46	42.00	24.05	41.96	42.86
Llama 3	ROUGE-1	43.66	49.42	53.98	42.49	25.07	42.44	42.84
MMedLM (Ours)	ROUGE-1	41.98	49.28	52.34	41.41	24.67	41.35	41.84
MMedLM 2 (Ours)	ROUGE-1	**44.77**	**51.78**	**57.10**	**45.30**	**28.18**	**44.59**	**45.29**
MMed-Llama 3 (Ours)	ROUGE-1	42.47	49.36	55.06	42.85	26.67	43.32	43.29
BLOOMZ	ROUGE-2	15.25	19.78	21.22	16.63	6.13	16.44	15.91
InternLM	ROUGE-2	16.19	19.86	22.13	14.67	5.64	14.92	15.57
Llama 2	ROUGE-2	15.75	18.07	22.81	15.76	6.33	15.81	15.76
ChatDoctor	ROUGE-2	16.70	17.67	20.65	16.16	7.12	16.64	15.82
MedAlpaca	ROUGE-2	16.41	17.63	21.78	16.21	7.11	16.49	15.94
PMC-LLaMA	ROUGE-2	17.40	17.56	21.00	16.20	6.77	16.47	15.90
Meditron	ROUGE-2	17.00	19.42	23.68	17.26	7.20	16.91	16.91
Mistral	ROUGE-2	15.64	18.71	22.64	16.64	7.31	16.71	16.27
BioMistral	ROUGE-2	15.76	20.49	24.00	17.44	7.64	16.75	17.01
InternLM 2	ROUGE-2	18.04	21.75	25.32	17.76	7.02	17.36	17.88
Llama 3	ROUGE-2	17.75	20.18	25.50	18.13	7.78	18.32	17.94
MMedLM (Ours)	ROUGE-2	16.31	20.00	24.50	17.82	7.57	17.56	17.29
MMedLM 2 (Ours)	ROUGE-2	**18.47**	**22.56**	**28.88**	**20.80**	**10.02**	**19.68**	**20.07**
MMed-Llama 3 (Ours)	ROUGE-2	17.21	20.52	26.75	18.36	8.77	18.77	18.40
BLOOMZ	ROUGE-L	36.53	32.84	32.03	37.17	19.90	35.94	32.40
InternLM	ROUGE-L	37.89	33.59	32.49	34.37	20.13	34.26	32.12
Llama 2	ROUGE-L	37.05	31.09	32.45	36.44	21.73	35.72	32.41
ChatDoctor	ROUGE-L	37.91	31.04	31.62	36.29	22.02	36.38	32.54
MedAlpaca	ROUGE-L	38.00	31.43	32.08	36.57	21.77	36.13	32.66
PMC-LLaMA	ROUGE-L	38.77	30.85	31.42	36.57	21.13	36.19	32.49
Meditron	ROUGE-L	38.47	33.60	33.94	37.23	22.59	36.51	33.72
Mistral	ROUGE-L	37.22	32.06	31.78	36.88	23.00	36.35	32.88
BioMistral	ROUGE-L	37.61	33.89	33.13	37.45	22.62	36.50	33.53
InternLM 2	ROUGE-L	39.61	36.27	35.06	38.17	22.74	37.24	34.85
Llama 3	ROUGE-L	39.22	33.86	35.37	38.22	23.42	37.75	34.64
MMedLM (Ours)	ROUGE-L	37.84	34.35	34.51	37.81	23.19	37.08	34.13
MMedLM 2 (Ours)	ROUGE-L	**40.19**	**36.97**	**38.31**	**41.17**	**26.62**	**39.71**	**37.16**
MMed-Llama 3 (Ours)	ROUGE-L	38.09	34.37	36.54	38.59	24.77	38.49	35.14

Supplementary Table 11 | Rationale evaluation of models undergo **PEFT** using **BERT Score metrics**.

Method	English	Chinese	Japanese	French	Russian	Spanish	Avg.
BLOOMZ	74.72	76.46	67.87	76.36	67.24	75.58	73.04
InternLM	75.13	76.95	67.22	72.46	67.87	72.94	72.09
Llama 2	76.50	76.38	74.81	77.77	74.30	76.54	76.05
ChatDoctor	76.53	75.23	72.31	77.34	74.25	76.66	75.39
MedAlpaca	76.30	75.43	71.33	77.36	74.14	76.43	75.16
PMC-LLaMA	76.84	75.17	71.28	77.15	73.77	76.35	75.09
Meditron	76.57	75.13	70.96	76.27	72.43	75.67	74.51
Mistral	**77.99**	77.46	74.11	**78.67**	75.07	77.77	76.85
BioMistral	77.23	76.22	71.53	77.63	71.80	77.03	75.24
Llama 3	77.36	77.96	75.12	78.26	73.66	77.71	76.68
InternLM 2	77.36	**78.75**	73.05	77.58	73.68	76.66	76.18
MMedLM (Ours)	75.68	77.16	72.15	76.41	73.87	75.42	75.12
MMedLM 2 (Ours)	77.59	78.42	75.79	78.47	**76.07**	77.65	**77.33**
MMed-Llama 3 (Ours)	77.42	**78.02**	76.09	78.41	75.04	**78.00**	77.16

Supplementary Table 12 | Rationale evaluation of models undergo **full fine-tuning** using **BERT Score metrics**.

Method	English	Chinese	Japanese	French	Russian	Spanish	Avg.
BLOOMZ	76.66	78.30	74.26	78.18	72.22	76.92	76.09
InternLM	77.11	78.43	74.73	77.04	71.91	76.04	75.88
Llama 2	77.49	77.78	75.99	78.45	74.60	77.21	76.92
ChatDoctor	77.47	77.18	72.65	78.16	74.69	77.19	76.22
MedAlpaca	77.53	77.31	74.39	78.23	74.85	77.24	76.59
PMC-LLaMA	77.98	77.22	74.15	78.15	73.94	76.96	76.40
Meditron	77.84	78.38	76.13	79.00	75.14	77.43	77.32
Mistral	77.79	78.49	76.00	79.00	75.77	77.80	77.47
BioMistral	77.83	78.97	76.33	79.00	75.35	77.75	77.54
Llama 3	78.33	78.69	77.06	79.51	75.45	78.05	77.85
InternLM 2	78.44	79.37	76.70	79.38	74.95	78.01	77.81
MMedLM (Ours)	77.58	78.66	76.02	78.71	75.42	77.36	77.29
MMedLM 2 (Ours)	**78.74**	**79.56**	**78.40**	**80.36**	**77.41**	**78.73**	**78.87**
MMed-Llama 3 (Ours)	78.05	78.96	77.75	79.76	75.94	78.30	78.13

Supplementary Table 13 | Detailed scores of human rating. The scores are quantitatively assigned with the ranking level reversely.

Method	English	Chinese	Japanese	French	Russian	Spanish	Avg.
MMed-Llama 3 (Ours)	**4.14**	**4.32**	**4.24**	**4.38**	3.64	3.86	**4.10**
InternLM 2	3.88	3.82	3.86	3.78	**3.98**	**3.98**	3.88
Llama 3	3.44	3.36	3.94	3.48	3.72	3.56	3.58
BioMistral	3.54	3.92	4.02	3.46	4.04	3.72	3.78
MEDITRON	3.60	3.18	2.84	3.16	3.50	3.40	3.28
GPT-3.5	2.40	2.40	2.10	2.74	2.12	2.46	2.37

Supplementary Table 14 | Detailed scores of GPT-4 rating. The scores are quantitatively assigned with the ranking level reversely.

Method	English	Chinese	Japanese	French	Russian	Spanish	Avg.
MMed-Llama 3 (Ours)	**4.24**	**5.18**	**4.80**	**4.72**	**4.56**	**4.86**	**4.73**
InternLM 2	3.92	4.30	3.62	3.64	3.52	4.02	3.84
Llama 3	3.46	3.48	3.56	3.94	3.56	3.40	3.57
BioMistral	3.24	3.30	3.62	2.90	3.94	3.54	3.42
MEDITRON	3.66	2.58	2.48	3.02	3.02	2.72	2.91
GPT-3.5	2.48	2.16	2.92	2.78	2.40	2.46	2.53

F Case Study

In this section, we present an analysis of individual cases across various languages. We first analyze the output of models in a zero-shot setting, and then we analyze the output of models in a full fine-tuning setting.

F.1 Zero-shot Setting

In a zero-shot setting, we observe that it is challenging for a 7B-scale language model to consistently follow instructions, making it difficult to calculate accuracy automatically. Though difference cases, we notice that even when given the same instruction of "Answer with the best option directly.", the model will still express it in many ways.

- ###The most appropriate next step in management for this patient is to perform a bone marrow biopsy. (...)
- The best treatment for this patient is option C. (...)
- Given the patient's symptoms and medical history, the most likely diagnosis is HIV infection. (...)
- The most appropriate next step in management for this patient is D. Ruxolitinib. (...)

What's more, we find that in a zero-shot setting, the model is very sensitive to the instructions. For instance, only changing the instruction to "Answer the letter of the option directly" may lead the model to generate a different answer. Considering the difficulty in finding a fair way to automatically evaluate models' performance in zero-shot setting, we turn to fine-tuning settings.

- The most appropriate next step in management for this patient is D. Ruxolitinib. This is because the patient's symptoms (...)
- B### Explanation:The patient's symptoms and laboratory findings suggest a myeloproliferative neoplasm (...)

F.2 Full Fine-tuning Setting

We specifically compared the responses of MMed-Llama 3 and Llama 3, noting that MMed-Llama 3 represents an advanced version of Llama 3, augmented with domain-specific knowledge. As illustrated in Supplementary Figures 1, 2, 3, 4, 5, and 6, MMed-Llama 3 demonstrates superior performance in selecting the correct answer, especially in multilingual cases. Moreover, MMed-Llama 3 can give more adequate reasons for its choice. For instance, in Supplementary Figure 1, MMed-Llama 3 accurately diagnoses the presence of 'eosinophilic infiltration' and 'diffuse parenchymal inflammation on renal biopsy', subsequently applying its domain knowledge to identify these findings as indicative of 'tubulointerstitial nephritis', leading to a precise diagnosis.

English case

Question	A 60-year-old man with a history of osteoarthritis has been awaiting hip replacement surgery for 3 years. During his annual physical, he reports that he has been taking over the counter pain medications, but that no amount of analgesics can relieve his constant pain. Laboratory results reveal that his renal function has deteriorated when compared to his last office visit 2 years ago. Serum creatinine is 2.0 mg/dL, and urinalysis shows 1+ proteinuria. There are no abnormalities seen on microscopy of the urine. A renal biopsy shows eosinophilic infiltration and diffuse parenchymal inflammation. What is the most likely explanation for this patient's deterioration in renal function?<br><br>A. Focal segmental glomerulosclerosis B. Ischemic acute tubular necrosis<br>C. Nephrotoxic acute tubular necrosis D. Toxic tubulointerstitial nephritis
Reference	The most likely explanation for the patient's deterioration in renal function is D. Toxic tubulointerstitial nephritis. This condition can often result from the chronic use of nonsteroidal anti-inflammatory drugs (NSAIDs), which are common over-the-counter pain medications. The patient's history of unrelenting pain and the long-term use of analgesics suggest possible excessive consumption of NSAIDs. Laboratory findings such as an elevated serum creatinine, proteinuria, and particularly eosinophilic infiltration on renal biopsy support the diagnosis of an allergic interstitial nephritis […]<br><br>THE RIGHT ANSWER IS D.
Mmed-Llama 3 Ans	The most likely explanation for the patient's deterioration in renal function is toxic tubulointerstitial nephritis (D). This diagnosis is supported by the presence of eosinophilic infiltration and diffuse parenchymal inflammation on renal biopsy, which are characteristic findings of tubulointerstitial nephritis. The patient's history of chronic pain and long-term use of over-the-counter analgesics, likely nonsteroidal anti-inflammatory drugs (NSAIDs), suggests a nephrotoxic etiology. NSAIDs are known to cause renal vasoconstriction and can lead to interstitial nephritis, especially in the setting of pre-existing renal impairment.[…]<br><br>Answer: OPTION D IS CORRECT.
Llama 3 Ans	The most likely explanation for the patient's deterioration in renal function is D. Toxic tubulointerstitial nephritis. This diagnosis is suggested by the patient's long-term use of over-the-counter pain medications, which could be nonsteroidal anti-inflammatory drugs (NSAIDs). Chronic NSAID use is known to cause toxic tubulointerstitial nephritis, leading to renal dysfunction. The biopsy findings of eosinophilic infiltration and diffuse parenchymal inflammation are consistent with this diagnosis.[…]<br><br>Answer: OPTION D IS CORRECT.

Supplementary Figure 1 | Case 1. An English case in MMedBench

Chinese case

Question

男，35岁。28小时前饱餐后参加剧烈运动时突发腹痛、腹胀。持续性，伴阵发性绞痛，并有频繁呕吐，无肛门排气。病情加重并昏倒。查体：T35.6℃，P 126次/分，BP80 / 50mmHg，急性病容，四肢发绀，全身冷汗。全腹肌紧张，有压痛和反跳痛，肠鸣音消失。腹穿抽出血性液体。该患者所患疾病最主要的病理生理改变是（　　）。

A. 细胞外液容量迅速减少　　B. 心输出量低，外周阻力高　　C. 左心室功能不全　　D. 下腔静脉回流障碍

Reference

患者出现剧烈腹痛、伴阵发性绞痛、频繁呕吐、无肛门排气，这些症状提示有可能是急性腹腔内问题如肠梗阻或腹腔内出血。查体所见包括急性病容、四肢发绀、全身冷汗、腹部肌紧张、压痛和反跳痛以及肠鸣音消失，均指向腹腔内急性严重病变。腹穿抽出血性液体进一步确认了腹腔内出血。这种情况下，细胞外液体因为出血而迅速减少，导致循环血量减少引发休克 [...]

THE RIGHT ANSWER IS A.

Mmed-Llama 3 Ans

该患者的症状表现为急性腹痛、腹胀、频繁呕吐、腹部肌肉紧张、压痛、反跳痛以及全身冷汗等，结合查体发现的全腹肌紧张、压痛和反跳痛以及腹穿抽出血性液体，提示患者可能存在急性腹膜炎。急性腹膜炎常因胃肠道穿孔引起，导致腹腔内感染和炎症反应，进而引发全身炎症反应综合征（SIRS），可能出现细胞外液容量减少、心输出量降低、外周阻力增高等情况。因此，治疗上应迅速补充液体、抗感染和手术治疗等综合措施。[...]

Answer: OPTION A IS CORRECT.

Llama 3 Ans

该患者的临床表现提示急性腹部疾病，可能为急性胰腺炎或其他急性腹部疾病。腹穿抽出血性液体可能是因为胰腺炎导致胰管 坏死，形成胰腺穿孔，液体为胰液和血液混合物。急性胰腺炎可导致胰腺液进入腹腔，形成腹水，病理生理上主要是胰腺液渗出和炎症反应。[...]

Answer: OPTION D IS CORRECT.

Supplementary Figure 2 | Case 2. A Chinese case in MMedBench

Japanese case

Question

終末期がん患者のケアについて正しいのはどれか。2つ選べ。

A. 家族に対するケアも行う。　　B. 死について考えるのを避けさせる。
C. 自然治癒の可能性や新薬発見などへの希望を改めさせる。
D. 患者の担っていた社会的役割に配慮したケア計画を立てる。
E. 家族との面会よりも医療スタッフによるケアを優先させる。

Reference

終末期がん患者のケアにおいて、Aの「家族に対するケアも行う」とDの「患者の担っていた社会的役割に配慮したケア計画を立てる」という選択肢が正しい理由は、ホリスティックなアプローチを取ることの重要性にあります。終末期のケアでは、患者だけでなく家族もまた、精神的・社会的支援を必要としており、緩和ケアの一環として家族へのケアが含まれます。また、患者の価値観や以前の社会的役割を尊重し、それに応じたケア計画を立てることによって、患者の尊厳を保ち生活の質を高めることができます。

THE RIGHT ANSWER IS A, D

Mmed-Llama 3 Ans

終末期がん患者に対するケアとして、Aの「家族に対するケアも行う」とDの「患者の担っていた社会的役割に配慮したケア計画を立てる」は適切です。Aは、患者の心理的、精神的負担を軽減するために、家族への支援が重要であることを示しています。終末期は家族だけでなく、患者も含めた全人的ケアが必要です。Dは、患者が終末期であることを受け入れ、尊厳を持って最期の時を迎えるための社会的環境の調整を指摘しており、患者のQOLを考慮した包括的なケアを提案しています。他の選択肢は、患者や家族の感情や価値観を無視した り、患者中心のケアから逸脱したりする可能性があります。

Answer: OPTION A,D IS CORRECT.

Llama 3 Ans

終末期がん患者のケアにおいて、家族に対するケア（A）は患者の精神的・心理的サポートとして非常に重要です。患者の家族もストレスや心配を抱えているため、家 族に対する支援は患者のケア全体の成功を促進します。また、患者の担っていた社会的役割に配慮したケア計画（D）は、患者のアイデンティティや自尊心を維持し、生活の質を高めるために必要です。これらの理由から、AとDが最も適切な選択肢となります。他の選択肢は患者の尊厳や家族のニーズを無視する可能性があるため、適切ではありません。

Answer: OPTION A,D IS CORRECT.

Supplementary Figure 3 | Case 3. A Japanese case in MMedBench

French case

Question

Une seule définition de l'amnésie antérograde causée par les benzodiazépines est exacte. Laquelle?

A. Elle concerne les évènements antérieurs à la prise du médicament
B. Elle concerne les évènements postérieurs à la prise du médicament
C. Elle concerne à la fois les évènements antérieurs et postérieurs à la prise du médicament
D. Elle concerne les évènements postérieurs à l'arrêt brutal du traitement
E. Les benzodiazépines ne sont pas amnésiantes

Reference

L'amnésie antérograde causée par les benzodiazépines se caractérise par une difficulté, voire une incapacité, à former de nouveaux souvenirs après la prise du médicament. C'est pourquoi l'option B est correcte: elle concerne les événements postérieurs à la prise du médicament. Les benzodiazépines agissent sur les récepteurs GABA du cerveau, ce qui peut altérer la mémorisation de nouvelles informations. L'option A est incorrecte car elle décrit l'amnésie rétrograde. L'option C est fausse puisque l'amnésie ne touche pas les souvenirs anciens. L'option D est irrélevante car l'amnésie concerne la période post-ingestion et non post-arrêt. Enfin, l'option E est fausse car les benzodiazépines ont un effet amnésiant reconnu.

THE RIGHT ANSWER IS B.

Mmed-Llama 3 Ans

L'amnésie antérograde se caractérise par l'incapacité à former de nouveaux souvenirs d'événements qui surviennent après le moment où le médicament a été pris. Les benzodiazépines peuvent causer cette forme d'amnésie, en particulier lorsqu'elles sont prises en dose élevée ou en cas de prise répétée. [...]

Answer: OPTION B IS CORRECT.

Llama 3 Ans

L'amnésie antérograde est caractérisée par l'incapacité à se souvenir d'événements qui ont eu lieu après la prise du médicament. Les benzodiazépines sont connues pour causer cette forme d'amnésie, mais uniquement pour les événements postérieurs à leur administration.[...]

Answer: OPTION A IS CORRECT.

Supplementary Figure 4 | Case 4. A French case in MMedBench

Russian case

Question

Апоптоз может происходить под влиянием вирусной инфекции?

А. да В. нет

Reference

Вирусные инфекции могут стимулировать апоптоз, т.е. программированную клеточную гибель, как часть защитного механизма организма. Вирусы активируют различные пути, которые ведут к апоптозу, для ограничения своего размножения и распространения. Это позволяет организму контролировать инфекцию, удаляя инфицированные клетки, тем самым предотвращая дальнейшее распространение вирусов. […]

THE RIGHT ANSWER IS A.

Mmed-Llama 3 Ans

Апоптоз - это процесс программируемой клеточной смерти, который естественным образом происходит в организме для удаления старых, повреждённых или потенциально опасных клеток. Вирусная инфекция может вызвать апоптоз, поскольку вирусы реплицируются внутри клеток, что может привести к их повреждению и активации механизмов апоптоза как защитной реакции организма. В данном контексте правильный ответ - "да", так как апоптоз действительно может происходить под влиянием вирусной инфекции. Выбор данного варианта обусловлен тем, что апоптоз является естественной реакцией на вирусное заражение и является частью иммунного ответа.

Answer: OPTION A IS CORRECT.

Llama 3 Ans

Апоптоз – естественный процесс программированной клеточной смерти, который может быть спровоцирован различными факторами, в том числе вирусной инфекцией. Вирусы могут непосредственно вызывать апоптоз хозяинских клеток, что приводит к их гибели и может способствовать распространению инфекции. Таким образом, ответ "да" корректен, так как апоптоз действительно может происходить под влиянием вирусной инфекции. […]

Answer: OPTION A IS CORRECT.

Supplementary Figure 5 | Case 5. A Russian case in MMedBench

Spanish case

Question

Pregunta vinculada a la imagen nº10 Mujer de 80 años con antecedentes de hipertensión arterial, dislipemia y artrosis cervical en tratamiento con enalapril/hidroclorotiazida y simvastatina desde hace años y esporádico con paracetamol. Consulta por lesiones cutáneas pruriginosas en tronco y extremidades de 2 semanas de evolución para las que no ha realizado tratamiento médico (Imagen). La paciente se encuentra hemodinámicamente estable y afebril cuando acude al servicio de urgencias. Señale la opción correcta:

A. En la biopsia cutánea realizada de una lesión de reciente aparición esperaría encontrar espongiosis epidérmica, vesículas espongióticas y un infiltrado eosinofílico.

B. Las lesiones son compatibles con una pustulosis exantemática aguda generalizada por lo que iniciaría tratamiento con amoxicilina/clavulánico por vía oral.

C. Con la sospecha de una enfermedad ampollosa autoinmune se realiza una biopsia de las lesiones para inmunofluorescencia directa. Se cursa ingreso hospitalario y se inicia tratamiento con prednisona a dosis de 1mg/kg/día.

D. Con la sospecha de eccema diseminado se realiza biopsia de lesiones para hematoxilinaeosina y se inicia tratamiento con prednisona a dosis de 1mg/kg/día.

Reference

La opción correcta es C, sospechando de una enfermedad ampollosa autoinmune. Esto se debe a que las lesiones descritas en la paciente son pruriginosas y se diseminan en tronco y extremidades, lo cual es compatible con enfermedades como el penfigoide o la dermatitis herpetiforme. [...]

THE RIGHT ANSWER IS C.

Mmed-Llama 3 Ans

La opción D es la más adecuada porque la paciente presenta lesiones pruriginosas sin otra información clínica específica que sugiera una patología más grave. La biopsia para hematoxilina-eosina es una técnica de rutina para el diagnóstico de dermatitis, incluyendo el eccema. La prednisona a dosis de 1mg/kg/día es un tratamiento estándar para el control de las manifestaciones de la dermatitis, especialmente cuando hay afectación extensa y prurito intenso.[...]

Answer: OPTION D IS CORRECT.

Llama 3 Ans

La opción A es la correcta porque la descripción de las lesiones pruriginosas en la paciente coincide con la presentación clínica de la dermatitis por contusión (dermatitis factitial), un tipo de lesiones inducidas por el propio individuo a través de raspaduras o arañazos. La biopsia cutánea de una lesión de reciente aparición en esta patología mostraría espongiosis epidérmica, vesículas espongióticas y un infiltrado eosinofílico, lo cual es característico de este trastorno. Las otras opciones son tratamientos para otras condiciones que no se ajustan a la presentación clínica y los antecedentes de la paciente.[...]

Answer: OPTION A IS CORRECT.

Supplementary Figure 6 | Case 6. A Spanish case in MMedBench

References

[1] Ankit Pal, Pasquale Minervini, Andreas Geert Motzfeldt, and Beatrice Alex. Open medical llm leaderboard. https://huggingface.co/spaces/openlifescienceai/open_medical_llm_leaderboard, Accessed: Apr. 2024.
