# Supplementary material for: Towards building multilingual language model for medicine
Source: Nat Commun. 2024 Sep 27;15:8384. doi: 10.1038/s41467-024-52417-z (PMC11436924; doi:10.1038/s41467-024-52417-z)
Supplement: Supplementary file 2 — Reporting Summary [file 41467_2024_52417_MOESM2_ESM.pdf]

nature portfolio

Corresponding author(s): Weidi Xie and Yanfeng Wang

Last updated by author(s): Jul 16, 2024

Reporting Summary

Nature Portfolio wishes to improve the reproducibility of the work that we publish. This form provides structure for consistency and transparency in reporting. For further information on Nature Portfolio policies, see our Editorial Policies and the Editorial Policy Checklist.

Statistics

For all statistical analyses, confirm that the following items are present in the figure legend, table legend, main text, or Methods section.

n/a	Confirmed	
☐	☒	The exact sample size (n) for each experimental group/condition, given as a discrete number and unit of measurement
☐	☒	A statement on whether measurements were taken from distinct samples or whether the same sample was measured repeatedly
☐	☒	The statistical test(s) used AND whether they are one- or two-sided<br>*Only common tests should be described solely by name; describe more complex techniques in the Methods section.*
☒	☐	A description of all covariates tested
☒	☐	A description of any assumptions or corrections, such as tests of normality and adjustment for multiple comparisons
☐	☒	A full description of the statistical parameters including central tendency (e.g. means) or other basic estimates (e.g. regression coefficient) AND variation (e.g. standard deviation) or associated estimates of uncertainty (e.g. confidence intervals)
☒	☐	For null hypothesis testing, the test statistic (e.g. F, t, r) with confidence intervals, effect sizes, degrees of freedom and P value noted<br>*Give P values as exact values whenever suitable.*
☒	☐	For Bayesian analysis, information on the choice of priors and Markov chain Monte Carlo settings
☒	☐	For hierarchical and complex designs, identification of the appropriate level for tests and full reporting of outcomes
☒	☐	Estimates of effect sizes (e.g. Cohen's d, Pearson's r), indicating how they were calculated

Our web collection on statistics for biologists contains articles on many of the points above.

Software and code

Policy information about availability of computer code

Data collection	The proposed datasets was collected by python 3.10.13.
Data analysis	The whole framework was implemented by python 3.10.13 and torch '1.13.0'. The code is available at https://github.com/MAGIC-AI4Med/MMedLM.

For manuscripts utilizing custom algorithms or software that are central to the research but not yet described in published literature, software must be made available to editors and reviewers. We strongly encourage code deposition in a community repository (e.g. GitHub). See the Nature Portfolio guidelines for submitting code & software for further information.

Data

Policy information about availability of data

All manuscripts must include a data availability statement. This statement should provide the following information, where applicable:

- Accession codes, unique identifiers, or web links for publicly available datasets
- A description of any restrictions on data availability
- For clinical datasets or third party data, please ensure that the statement adheres to our policy

MMedC is available at https://huggingface.co/datasets/Henrychur/MMedC.
MMedBench is available at https://huggingface.co/datasets/Henrychur/MMedBench.
MedQA is available at https://github.com/jind11/MedQA.

IgakuQA is available at https://github.com/jungokasai/IgakuQA.
FrenchMedMCQA is available at https://github.com/qanastek/FrenchMedMCQA.
RuMedDaNet is available at https://github.com/pavel-blinov/RuMedBench/tree/main/data/RuMedDaNet.
Head-QA is available at https://github.com/aghie/head-qa
PubMedQA is available at https://pubmedqa.github.io/.
MedMCQA is available at https://github.com/medmcqa/medmcqa.
MMLU is available at https://github.com/hendrycks/test.

Research involving human participants, their data, or biological material

Policy information about studies with human participants or human data. See also policy information about sex, gender (identity/presentation), and sexual orientation and race, ethnicity and racism.

Reporting on sex and gender	This is not relevant to our study.
Reporting on race, ethnicity, or other socially relevant groupings	This is not relevant to our study.
Population characteristics	This is not relevant to our study.
Recruitment	This is not relevant to our study.
Ethics oversight	This is not relevant to our study.

Note that full information on the approval of the study protocol must also be provided in the manuscript.

Field-specific reporting

Please select the one below that is the best fit for your research. If you are not sure, read the appropriate sections before making your selection.

☒ Life sciences ☐ Behavioural & social sciences ☐ Ecological, evolutionary & environmental sciences

For a reference copy of the document with all sections, see nature.com/documents/nr-reporting-summary-flat.pdf

Life sciences study design

All studies must disclose on these points even when the disclosure is negative.

Sample size	For the all the datasets used in this paper, we strictly follow the official train-test split of public datasets to ensure fair comparisons and reproducible results. The only exception is IgakuQA, which has no official train-test split. So we randomly split the train-test dataset.
Data exclusions	Data were not excluded from analysis.
Replication	Our experiment is carried out by programmers. We release all the codes to make sure all results are reproducible.
Randomization	In our experiment, for the public available datasets, we strictly follow the official train-test split. The only exception is IgakuQA, which has no official train-test split. So we randomly split the train-test dataset for this dataset when constructing the proposed MMedBench.
Blinding	In our human rating evaluation, the raters were blind to the source of the response.

Reporting for specific materials, systems and methods

We require information from authors about some types of materials, experimental systems and methods used in many studies. Here, indicate whether each material, system or method listed is relevant to your study. If you are not sure if a list item applies to your research, read the appropriate section before selecting a response.

Materials & experimental systems

n/a	Involved in the study	
☒	☐	Antibodies
☒	☐	Eukaryotic cell lines
☒	☐	Palaeontology and archaeology
☒	☐	Animals and other organisms
☒	☐	Clinical data
☒	☐	Dual use research of concern
☒	☐	Plants

Methods

n/a	Involved in the study	
☒	☐	ChIP-seq
☒	☐	Flow cytometry
☒	☐	MRI-based neuroimaging
